# Comparison between a conventional tool and deep learning models for RNA velocity analysis of scRNA-Seq data

**DOI:** 10.1007/s00438-026-02429-9

**Published:** 2026-05-13

**Authors:** Matheus Rodrigues Sauda, Ana Beatriz Rodrigues, Maria Letícia de Oliveira Lyra, Rejane Maria Tommasini Grotto, Lei Gu, Tatiana de Campos Melo, Guilherme Targino Valente

**Affiliations:** 1 Laboratory of Applied Biotechnology, São Paulo State University, Botucatu, 18618-687 Sao Paulo State Brazil; 2 Nucleus of Artificial Intelligence of Clinical Hospital of Medical School of Sao Paulo State University, São Paulo State University, Botucatu, 18618-687 Sao Paulo State Brazil; 3 Present Address: Department of Plant Protection, Sāo Paulo State University, Botucatu, 18610-034 Sāo Paulo State Brazil; 4 Max Planck Institute for Heart and Lung Research, Ludwigstraße number 43, Bad Nauheim, 61231 Hessen Germany; 5 Nucleus of Urgency and Emergency of Clinical Hospital of Medical School of Sao Paulo State University, São Paulo State University, Botucatu, 18618-687 Sao Paulo State Brazil; 6 Department of Pediatric, São Paulo State University, Botucatu, 18618-687 Sao Paulo State Brazil

**Keywords:** Single-cell RNA-Seq, Benchmark, RNA velocity, Deep-learning

## Abstract

Single-cell RNA sequencing (scRNA-Seq) enables analysis of gene expression at single-cell resolution. RNA velocity analysis infers the temporal dynamics of transcriptional states from the relative abundances of spliced/unspliced mRNA quantified via scRNA-Seq. Classical RNA velocity approaches, such as scVelo, implement gene-specific kinetic modeling. Deep learning methods including DeepVelo, VeloVI, LatentVelo, SymVelo, and scTour are based on variational autoencoders (VAEs), which allow to enhance the robustness and accuracy by leveraging nonlinear latent representations. Here, we systematically evaluated the performance of deep learning RNA velocity tools by comparing with the scVelo dynamical model to access the possible advantages of VAE-base methods. For this purpose, public datasets (GSE149689 and GSE203233) were initially processed using a standard scRNA-Seq pipeline. Comparisons among results of selected velocity tools were conducted using cosine similarity of velocity vectors to assess directional concordance, and by mean squared error analysis of trajectory continuity for the deep learning models. Overall, VAE methods produced significant, richer, and more directionally coherent and consistent velocity fields than the classical model. Our findings indicate that deep learning models provide more consistent and biologically plausible cell-state trajectories, although at the expense of higher computational demands and reliance on accurate splicing quantification. Altogether, our results underscore the relevance of VAE-based frameworks to advance RNA velocity analysis while highlighting the need for careful preprocessing.

**Supplementary Information:**

The online version contains supplementary material available at 10.1007/s00438-026-02429-9.

## Introduction

Single-cell RNA sequencing (scRNA-Seq) is a powerful technique to assess gene expression at the individual cell level (Chen et al. 2019; Schultheis et al. 2025), allowing the observation of tissue heterogeneity not clear in classical bulk transcriptome analysis (AlJanahi et al. 2018). In addition to capturing average expression profiles, scRNA-Seq allows us to discover novel cell types and define cellular subtypes within a given tissue (AlJanahi et al. 2018).

The scRNA-Seq workflow begins with reverse transcription of RNA to cDNA, followed by amplification and sequencing of complementary DNA using next-generation sequencing (NGS) platforms (Saliba et al. 2014). However, technical noise and biological variability, such as stochastic transcription events characterized by random activation or inactivation of genes, scRNA-Seq data analysis requires multiple processing steps to ensure that results are robust and reproducible (Chen et al. 2019).

**Fig. 1:**
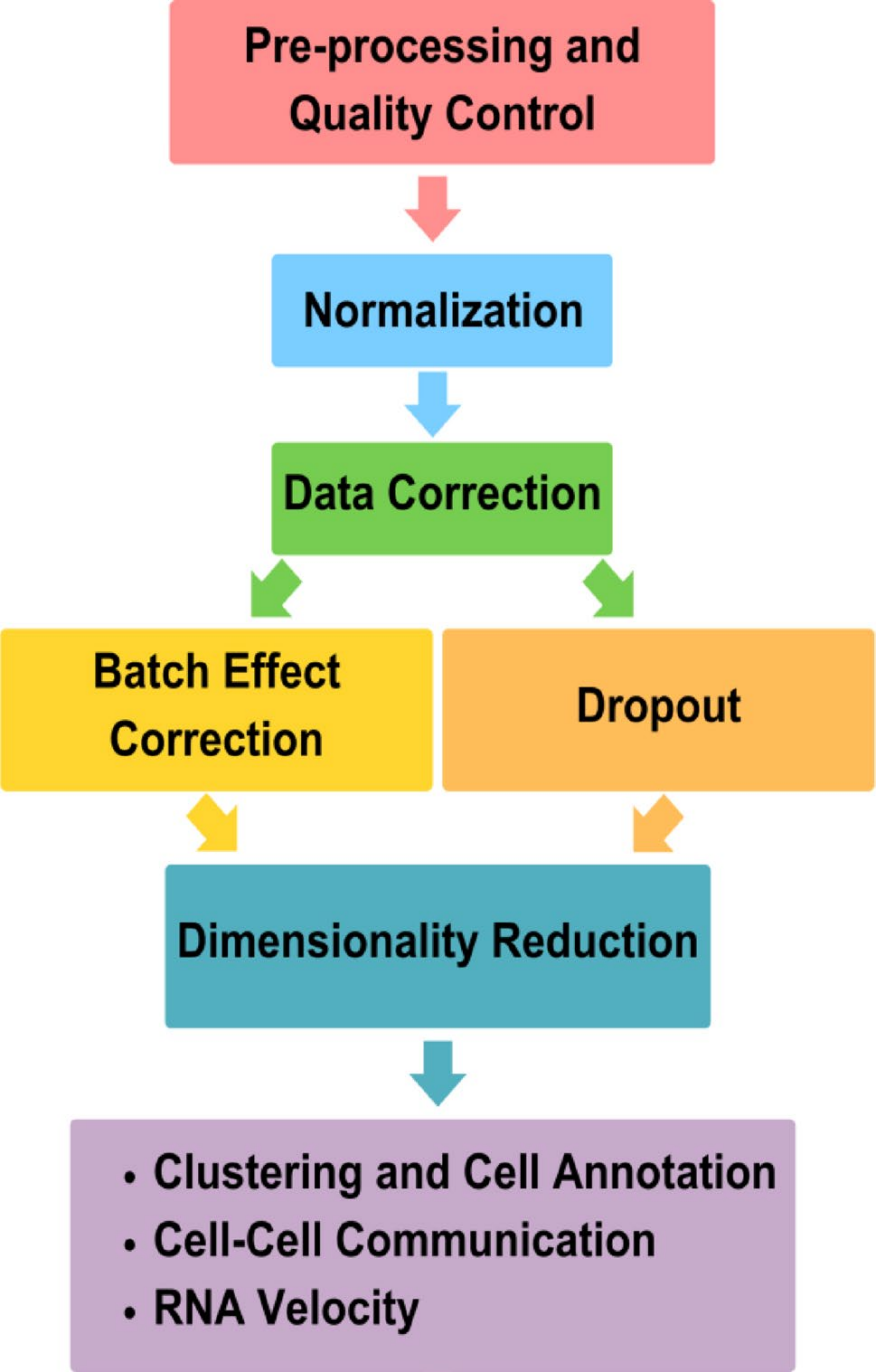
General workflow for scRNA-Seq analysis. The pipeline includes pre-processing and quality control, normalization, and data correction. The corrected data are processed through dimensionality reduction, followed by downstream analyzes such as clustering and cell annotation, cell–cell communication, and RNA velocity. (adapted from (Erfanian et al. 2023).

The scRNA-Seq pipeline (Fig. 1) clarifies each processing step and fosters the development of specialized tools tailored to each phase. Usually, the workflow begins with pre-processing and quality control to remove erroneous or missing cell barcodes and to address the higher complexity and noise inherent to scRNA-Seq when compared to bulk RNA-Seq data (Kebschull and Zador 2018; Erfanian et al. 2023). The data is then normalized to correct sample-to-sample variation and to reduce biases introduced by technical or biological artifacts (Erfanian et al. 2023). Subsequently, the data correction is performed that includes dropout removal and batch-effect correction to deal with false zeros from inefficient mRNA capture and technical variability introduced by different instruments, laboratories, or operators (Erfanian et al. 2023). The next step is to reduce the dimensionality of the gene-expression matrices to transform noisy and high dimensional data into a more compact representation appropriate for downstream analyses (Sun et al. 2019).

RNA velocity is an extension of scRNA-Seq analysis to infer the temporal dynamics of gene expression at a single-cell resolution (Zheng et al. 2017; La Manno et al. 2018). The concept of RNA velocity deeply influenced the study of single-cell transcriptomics by allowing the characterization of static cellular states and the prediction of cellular trajectories during processes such as differentiation, development, and response to stimuli (Bergen et al. 2021).

RNA velocity analysis is based on the quantification of a ratio between spliced/unspliced mRNA transcripts. This step allows to model the rate of transcript abundance changes by assuming that unspliced mRNAs level reflect newly transcribed molecules, while spliced mRNAs level represent more mature transcripts (Bergen et al. 2021; Zheng et al. 2023). A positive RNA velocity value indicates gene upregulation when the observed abundance of unspliced mRNA exceeds the expected steady-state level. Conversely, a negative velocity suggests downregulation when the amount of unspliced transcripts falls below the expected steady-state level (Bergen et al. 2020).

Two initial computational frameworks were developed to estimate the RNA velocity. Velocyto (La Manno et al. 2018) computes the RNA velocity as a time derivative of the splicing ratio using linear regression. This approach assumes a constant rate of splicing between genes and the presence of a partial steady-state expression level in at least one cell within the sample (Bergen et al. 2021). Although Velocyto is based on simple assumptions, it founded subsequent methods and remains a common source of splicing-aware count matrices used by downstream tools such as scVelo.

scVelo overcomes the limitations of the Velocyto model to deal with heterogeneous cell populations. In this case, scVelo infers gene-specific transcription, splicing, and degradation rates, and a shared latent time assumed as an “internal clock”, positioning each cell along its underlying biological trajectory. scVelo achieves more accurate and robust velocity estimates compared to earlier approaches by incorporating stochastic modeling of transcriptional and post-transcriptional events (Bergen et al. 2020).

Deep learning is a subfield of machine learning and employs deep neural networks (DNNs) to model complex data patterns with high accuracy (Jim Holdsworth and Mark Scapicchio 2025; Paluszek and Thomas 2020; Kufel et al. 2023; Erfanian et al. 2023). Recurrent Neural Networks (RNNs) are effective for sequential data, while Convolutional Neural Networks (CNNs) specialize in image analysis. Generative Adversarial Networks (GANs) generate realistic synthetic data through a generator-discriminator framework. Autoencoders (AEs) and their extension Variational Autoencoders (VAEs) are applied in unsupervised learning to reduce dimensionality, data integration, and generate realistic data variations (Kufel et al. 2023; Erfanian et al. 2023). VAEs play a crucial role in the development of methods for scRNA-Seq analysis, such as RNA velocity, due to their ability to model heterogeneous and high-dimensional data (Paluszek and Thomas 2020; Kufel et al. 2023). Autoencoder-based approaches are effective in extracting biologically meaningful insights from complex scRNA-Seq datasets. Altogether, VAE approaches can enhance the resolution of biologically meaningful transitions in heterogeneous single-cell datasets (Sharma et al. 2021; Janiesch et al. 2021), as demonstrated by marker-informed concordance and functional enrichment of trajectory-driving genes in the COVID-19 subgroup (Roberts and Barb 2018; Silvestre-Roig et al. 2019; Ravenhill et al. 2020; Cormican and Griffin 2020; Van Faassen et al. 2021). Finally, DeepVelo (Chen et al. 2022), VeloVI (Gayoso et al. 2024), LatentVelo (Farrell et al. 2023), SymVelo (Xie et al. 2024) and scTour (Li 2023) used for RNA velocity illustrate the potential of VAEs to improve the accuracy and robustness of cell state trajectory inference.

Despite progress in the application of VAEs in RNA velocity analysis from scRNA-Seq data, a suitable predictive comparison among these tools and comparisons to a traditional tool are not currently available. Therefore, the goal of this study was to systematically evaluate the performance of RNA velocity analysis based on deep learning approaches and to compare their performance to that of a classical methodology. Furthermore, we evaluated the biological plausibility of the RNA velocity vectors inferred through these approaches. Overall, VAE methods produced better results than the classical model, underscoring the potential of VAE-based frameworks to advance RNA velocity analysis. Finally, we also presented the best practices for a proper scRNA-Seq velocity analysis.

## Methods

### Dataset selection

A literature review and a systematic search were performed in the GEO (Gene Expression Omnibus) database using the keywords ‘single-cell’, ‘influenza’ and ‘human’, leading to the selection of the dataset GSE149689. This dataset contains single-cell RNA-seq profiles of 16 human PBMC samples, including healthy controls, patients with influenza, and patients with COVID-19. Overall, this dataset was used in studies to investigate the immune mechanisms underlying the progression of severe COVID-19, highlighting hyper-inflammatory signatures compared to influenza (Seok Lee et al. 2020; Choi et al. 2022). Additionally, a mouse scRNA-Seq dataset was selected using the keywords ‘mus musculus’ and ‘single-cell’, resulting in GSE203233. This dataset consists of six larynx samples from wild-type C57BL/6J mice, including three conventionally raised (ConvR) and three germ-free (GF) animals. This dataset was previously analyzed in the context of generating a single-cell atlas of the upper airway and assessing how the commensal microbiota influence the differentiation of immune and epithelial cells (An et al. 2024).

### Data preprocessing and quality control

Raw sequencing files in FASTQ format for both datasets were downloaded from the ENA (European Nucleotide Archive) (Harrison et al. 2021). All FASTQ files were preprocessed and aligned against their appropriate reference genomes using Cell Ranger v8.0.1. This step included quality filtering, alignment to reference genomes (GRCh38 for human and GRCm39 for mouse), and quantification of gene expression at a single-cell level. The resulting BAM files were used for downstream analysis in Velocyto to quantify spliced and unspliced transcripts.

Data preprocessing was performed in Scanpy v1.1 in a Jupyter Notebook v7.4.4 environment. For this purpose, the raw 10X outputs were loaded into individual Scanpy AnnData objects. The 16 samples from the GSE149689 dataset were grouped into influenza, control, and COVID-19 classes. The samples from the GSE203233 dataset were divided into conventionally raised (ConvR) and germ-free (GF) groups. Quality-control metrics were computed using the pp.calculate_qc_metrics(). Mitochondrial genes were identified and cells with < 200 or > 30% of mitochondrial genes were excluded. Genes expressed in < 3 cells were also removed. Additional outlier filtering of library complexity was performed by excluding cells outside the 2nd-98th percentiles considering the gene counts.

Data were normalized and logarithmically transformed. Subsequent analysis was performed using highly variable genes (HVGs) (pp.highly_variable_genes with min_mean = 0.0125, max_mean = 3, min_disp = 0.5). The effects of total gene counts and the percentage of mitochondrial genes were removed to minimize technical bias, and gene expression values were Z-scaled (max_value = 10) to limit the influence of extreme values. Dimensionality reduction was performed using PCA, neighborhood graphs were calculated (n_neighbors = 20 and n_pcs = 6), and the cluster structure was inferred using the Leiden algorithm. UMAP was used for visual inspection of reduced two-dimensional data.

### Cell type annotation, and RNA velocity inference

Cell type annotation was performed with CellTypist v1.7.1 using the pre-trained immune model Immune_All_Low.pkl. The predicted labels were recorded as “dominant cell type” and used to assist cluster interpretation.

For RNA-velocity analyses, Velocyto loom outputs were merged with Loompy and integrated into the corresponding AnnData objects to allow both spliced and unspliced layers to be available for downstream inference. Then, we compared the velocity results from the scVelo, and DeepVelo, VeloVI, and LatentVelo VAE models.

As a classical baseline, the RNA velocity was estimated with scVelo v0.3.3. The same filtering criteria mentioned were applied, including selection of the top 2,000 HVGs and exclusion of low-expressing genes (< 20 counts). Statistical moments (mean and variance of gene counts across adjacent cells) were computed using scv.pp.moments() prior to velocity estimation to inform gene transcription dynamics. Transcriptional kinetics and cell-state transitions were inferred using the dynamical model of scVelo (scv.tl.recover_dynamics() and scv.tl.velocity(mode=’dynamical’)). For deep-learning approaches, identical quality control, normalization, and gene-selection procedures mentioned were applied prior to training the DeepVelo, VeloVI, and LatentVelo models. Each model incorporated temporal information from spliced and unspliced expression profiles to infer RNA velocity vectors.

### Model training details

For deep learning training and velocity inferences using DeepVelo, VeloVI, and LatentVelo, we utilized the default network architectures and training hyperparameters (latent dimensions, batch sizes, learning rates, and training epochs) provided by their libraries. This approach was chosen to evaluate the algorithms under their standard recommended usage conditions, avoiding bias from our personal adjustments. Furthermore, we preserved the automated built-in features, such as validation-based early stopping for VeloVI and the dynamic scaling of the ‘coeff_s’ parameter according to the dataset’s spliced reads ratio for DeepVelo.

The LatentVelo model was executed in Google Colab with T4 GPU acceleration, whereas other tools were executed using a local CPU resource (Dell Server Precision with 16 cores from Intel Xeon, 128 Gb of RAM, 5 Tb of hard drive, Ubuntu 24.04 operational systems and without GPU). We assessed the computational footprint demands including execution time and peak RAM consumption across datasets.

### Statistical analysis for benchmarking, and data visualization

For comparative assessment, velocity outputs from DeepVelo, VeloVI and LatentVelo were benchmarked against the scVelo baseline. Confidence in inferred velocity vectors was quantified using cosine similarity, and distributions of similarity scores (restricted to non-zero values to avoid bias from null estimates) were statistically compared using the two-sided Mann-Whitney U test. Direct pairwise cosine similarity comparisons were also performed among deep learning models. Additionally, distributions of mean squared error (MSE) in cellular continuity were computed and plotted to further characterize differences between deep learning methods. To further evaluate the local consistency of the inferred vector fields, we computed the cluster-wise velocity coherence scores for each model. Furthermore, internal consistency of inferred vector fields was assessed through velocity consistency scores using scv.tl.velocity_confidence; this metric measures the local alignment of velocity vectors with their nearest neighbors, allowing for a quantitative performance benchmark across human and mouse datasets.

RNA-velocity models assume that the ratio of spliced/unspliced reads reflects true transcriptional kinetics. We accessed the robustness of the models analyzed and the reliability of the threshold of the velocity vectors to low unspliced counts by a downsampling experiment. For this purpose, we performed a binomial downsampling on unspliced mRNA content (75%, 50%, and 25% of the original data) using the Covid-19 subgroup as model. The results of each framework (scVelo, DeepVelo, VeloVI, and LatentVelo) were compared by analyzing the stability of velocity coherence scores across the mentioned decreasing fractions.

To biologically validate inferred trajectories within key immune populations (CD16 + NK cells, classical monocytes, and neutrophils), the top velocity-driving genes ranked by their absolute velocity scores were extracted from targeted clusters of Human Covid-19 samples (part of GSE149689 dataset) using the scv.tl.rank_velocity_genes function. Functional enrichment analysis of these genes was performed using the g: Profiler v1.55.0 (Raudvere et al. 2019) against the Gene Ontology Biological Process (Ashburner et al. 2000), KEGG (Kanehisa and Goto 2000), and Reactome (Milacic et al. 2024) databases.

All velocity inferences obtained here were visualized as stream plots overlaid on UMAPs embeddings, allowing a direct visual comparison of transition graphs across “dominant cell type” clusters. Additional visualizations (histograms, density plots, dotplots, and other UMAPs) were used to summarize comparisons between DeepVelo, VeloVI, LatentVelo, and the scVelo framework. Functional enriched terms (p-value < 0.05) were visualized as dotplots.

## Results

**Fig. 2:**
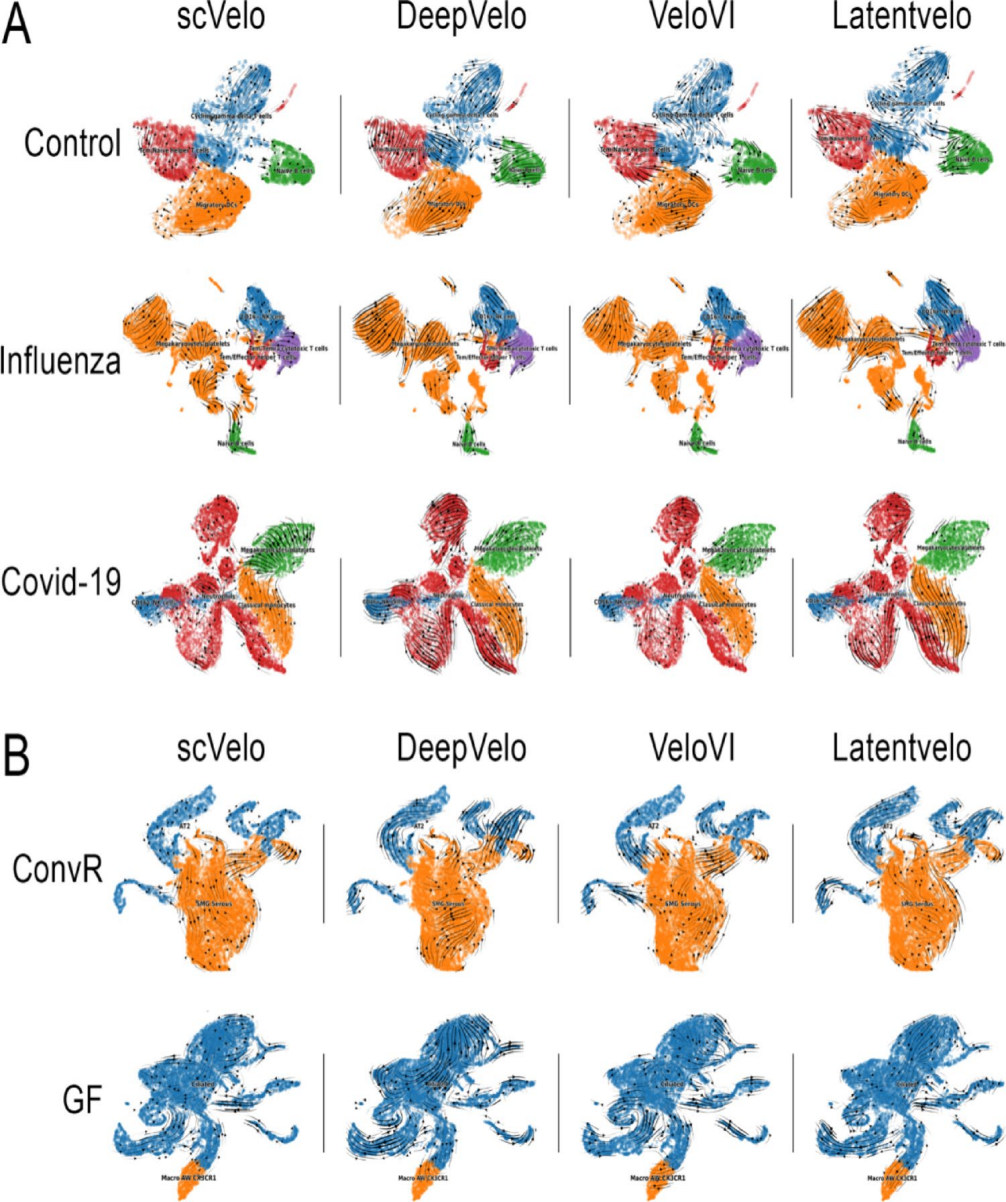
Inferred RNA velocity for the human and *Mus musculus* datasets. RNA velocity fields were inferred for the GSE149689 (**A**) dataset across the control, influenza, and COVID-19 groups, and for the GSE203233 (**B**) dataset across the conventionally raised (ConvR) and germ free (GF) groups using four methods: scVelo, DeepVelo, VeloVI, and LatentVelo

The convergence arrows in the plot of the inference of the RNA velocity highlight regions of transcriptional similarity, while the divergent arrows indicate potential differentiation or transitions between cellular states: these arrows illustrate the dynamic trajectories underlying the cellular landscape in each condition. The RNA velocity inferred for GSE149689 (human dataset) across control, influenza, and COVID-19 groups using scVelo, DeepVelo, VeloVI, and LatentVelo showed that cells with similar transcriptional states formed converging regions in the vector field, while divergent arrows highlighted areas of potential differentiation under all conditions. These patterns reflect the inferred progression of cells through various transcriptional states (Fig. 2A). Similarly, Conventionally Raised (ConvR) and germ-free (GF) groups of GSE203233 (mouse dataset) showed coherent directional flows between cellular states (Fig. 2B).

**Fig. 3:**
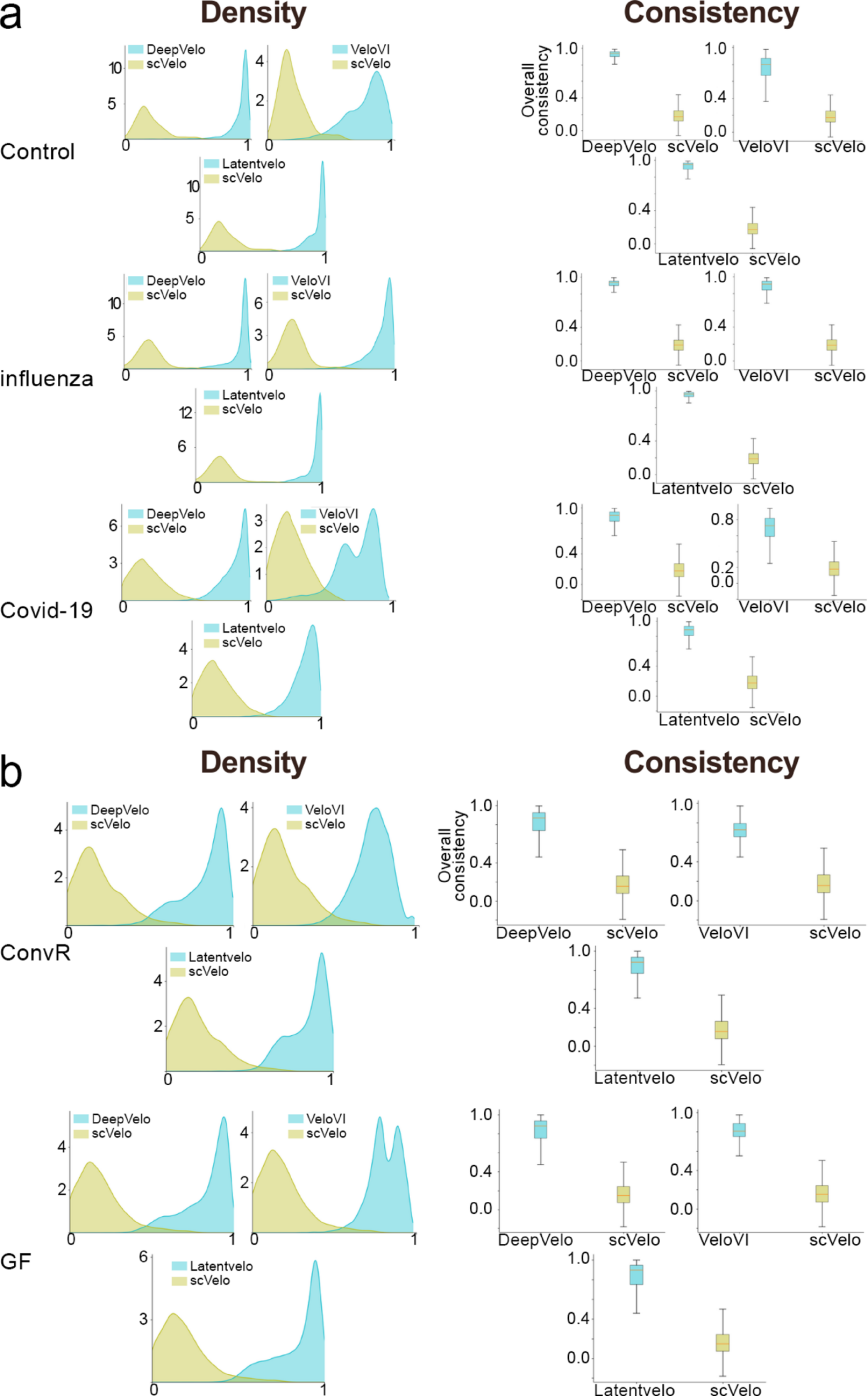
Cosine similarity plots for the human and *Mus* *musculus* datasets. Cosine similarity plots were generated from the RNA velocity vectors inferred by each computational tool for the GSE149689 dataset (**A**) across control, influenza, and COVID-19 groups and for the GSE203233 dataset (**B**) across conventionally raised (ConvR) and germ-free (GF) groups. X-axis: cosine similarity values; Y-axis: relative frequency (density) of each value. Peaks near 1.0 reflect strong directional agreement and biologically consistent transitions, whereas broader distributions or peaks near 0 indicate greater variability and less concordance between inferred trajectories

The cosine similarity was computed between the velocity vectors inferred by each tool to quantify the degree of alignment in the RNA velocity directions independently of their magnitude. This metric provides a measure of concordance between models, enabling a direct comparison of how similar the inferred cellular transitions are between methods. The comparison among RNA velocity strategies here analyzed showed that deep learning (DeepVelo, LatentVelo, and VeloVI) approaches produced higher values of cosine similarity, indicating more consistent velocity directions compared to the classical method (scVelo). We highlight that the LatentVelo and DeepVelo results seem to be more closely related (Figs. 3A-B).

We used the Mann-Whitney U test to statistically evaluate whether the distributions of cosine similarity values, interpreted as a measure of confidence in the inferred directions, differed significantly between methods. The results demonstrated statistically significant differences (p-value < 10^− 15^) between all groups in both datasets (Table 1).

**Table 1:** Results of the Mann-Whitney U test for the human and Mus musculus datasets

Group	Dataset	Mann-Whitney U Test	*p*-value	Deep Learning tool
COVID-19	A	173,622,946	< 10^− 15^	VeloVI
COVID-19	A	177,990,589	< 10^− 15^	Deepvelo
COVID-19	A	177,960,452	< 10^− 15^	LatentVelo
Influenza	A	132,982,613	< 10^− 15^	VeloVI
Influenza	A	133,028,868	< 10^− 15^	DeepVelo
Influenza	A	133,073,839	< 10^− 15^	Latentvelo
Control	A	35,256,343	< 10^− 15^	VeloVI
Control	A	35,477,146	< 10^− 15^	Deepelo
Control	A	35,509,230	< 10^− 15^	LatentVelo
ConvR	B	155,065,740	< 10^− 15^	VeloVI
ConvR	B	154,717,151	< 10^− 15^	Deepvelo
ConvR	B	154,834,155	< 10^− 15^	LatentVelo
GF	B	89,054,315	< 10^− 15^	VeloVI
GF	B	89,220,555	< 10^− 15^	Deepvelo
GF	B	89,461,915	< 10^− 15^	LatentVelo

Pairwise Mann-Whitney U tests were conducted to compare the distributions of RNA velocity values obtained with scVelo against DeepVelo, LatentVelo, and VeloVI. Results are shown for the GSE149689 human dataset (A), including control, influenza, and COVID-19 groups, and for the GSE203233 *Mus musculus* dataset (B), including conventionally raised (ConvR) and germ-free (GF) groups. Statistically significant p-values (< 10^− 15^) indicate relevant differences between methods

**Fig. 4:**
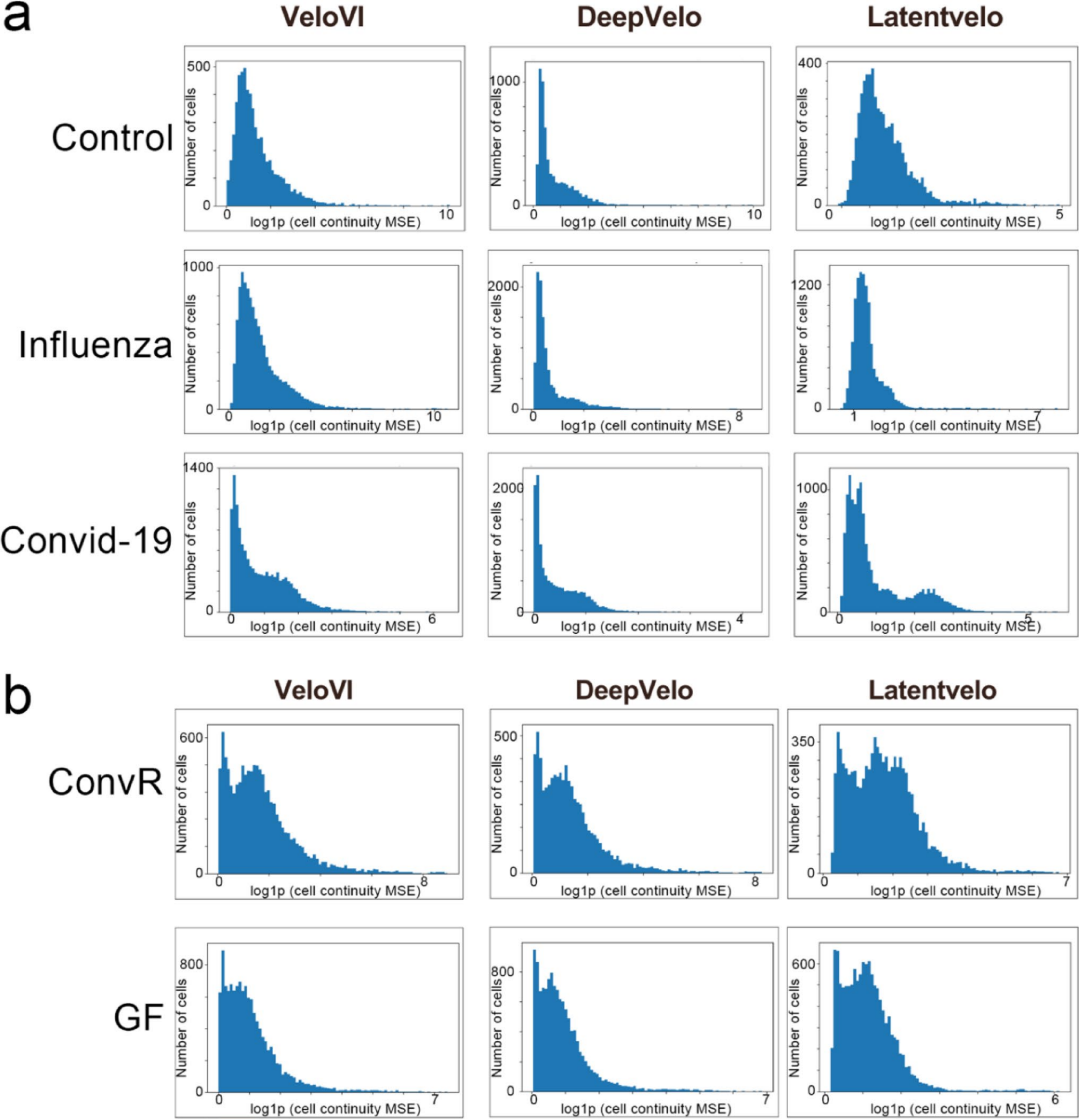
Histograms of MSE log-transformed in cellular continuity for human and *Mus musculus* datasets. Histograms of MSE log-transformed in cellular continuity were generated for the GSE149689 dataset (control, influenza, and COVID-19 groups) (**A**) and the GSE203233 dataset (conventionally raised (ConvR), and germ-free (GF) groups) (**B**) across all deep learning-based RNA velocity tools analyzed. Lower log(MSE) values indicate greater continuity, reflecting consistent and biologically plausible transitions. Higher log(MSE) values denote discontinuities in the inferred trajectories

Although cosine similarity showed that LatentVelo and DeepVelo were closely aligned, the mean squared error (MSE) analysis used to quantify the uniformity of the velocity trajectories across the cellular manifold indicated that DeepVelo reached lower errors, suggesting more coherent velocity predictions compared to LatentVelo (Fig. 4A-B).

**Fig. 5:**
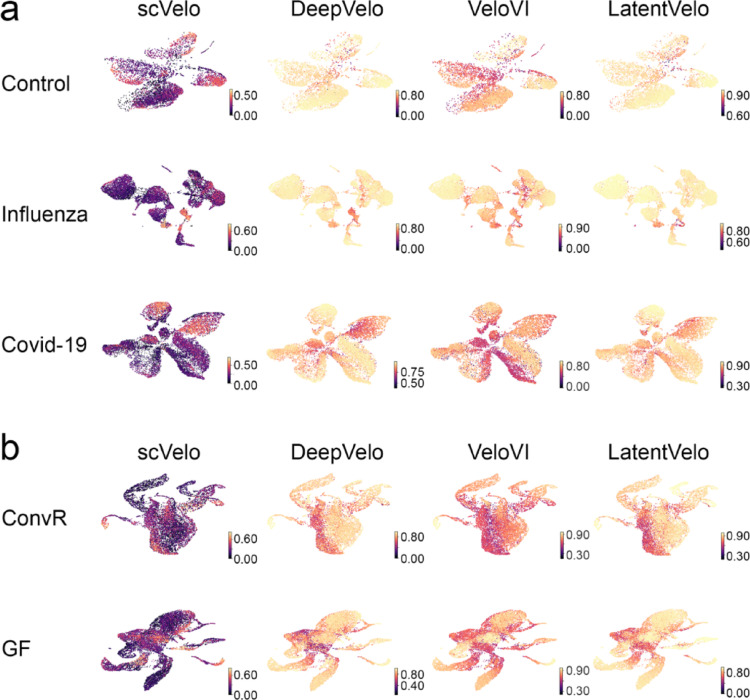
Cluster-wise velocity coherence score analysis for human and *Mus musculus* datasets. Data for the GSE149689 dataset (control, influenza, and COVID-19 groups) (**A**) and the GSE203233 dataset (conventionally raised (ConvR), and germ-free (GF) groups) (**B**) in scVelo and all deep learning–based RNA velocity tools analyzed. Higher score values indicate more reliable arrow directions inferred by the tool, likely representing a real biological process

To further validate the local consistency of the velocity predictions, we analyzed the cluster-wise velocity coherence scores. Deep learning models consistently exhibited higher directional coherence within the main biological populations compared to the classical scVelo baseline, which presented lower coherence scores across all conditions evaluated. LatentVelo, and DeepVelo achieved the highest overall coherence: LatentVelo produced the most highly aligned vector fields in most of subgroups, with a peak > 0.96 in Control and Influenza human subgroups (Table 2, and Fig. 5).

**Table 2:** Cluster-wise velocity coherence scores across groups

Group	ScVelo	DeepVelo	LatentVelo	VeloVI
Covid-19	0.192304	0.888273	0.879840	0.637920
Influenza	0.183026	0.920831	0.961263	0.874419
Control	0.198547	0.915520	0.963017	0.743616
ConvR	0.192288	0.789035	0.815597	0.672400
GF	0.193351	0.808475	0.840170	0.770000

The values represent the directional coherence of the inferred RNA velocity vectors for each experimental condition across the evaluated models. Coherence scores range from 0 to 1, where values closer to 0 and 1.0 indicate random or noisy vector orientations and highly aligned, robust, and locally consistent vector fields within biological clusters, respectively

**Fig. 6:**
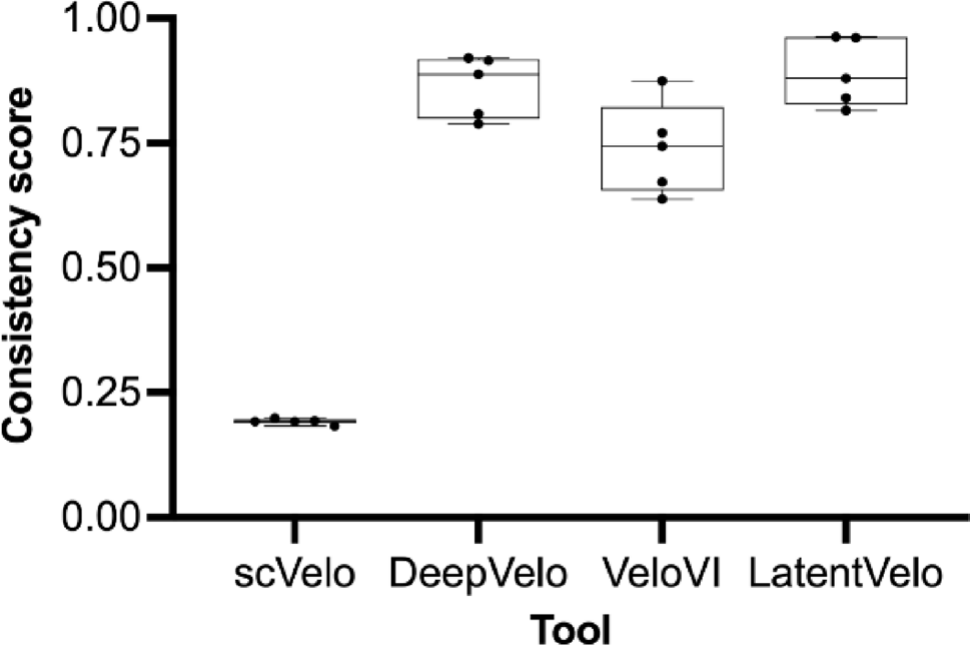
Cluster-wise velocity consistency score across tools analyzed. Consistency score range from 0 to 1, where values closer to 0 and 1.0 indicate random or noisy vector orientations and highly aligned, consistent, and locally stable vector fields within biological clusters, respectively (details of values per sample are presented in the Supplementary Table 1)

Comparative benchmarking based on consistency score across all samples showed that the deep learning tools analyzed here significantly improved vector field consistency over scVelo. Although the consistency score of scVelo remained constantly low (~ 0.19), LatentVelo and DeepVelo provided the highest values of consistent trajectories (Fig. 6), particularly in human samples Control and Influenza (scores peaked > 0.9). DeepVelo also showed a very superior score (~ 0.88) in the Covid-19 subgroup than VeloVI (~ 0.63) (Supplementary Table 1).

**Fig. 7:**
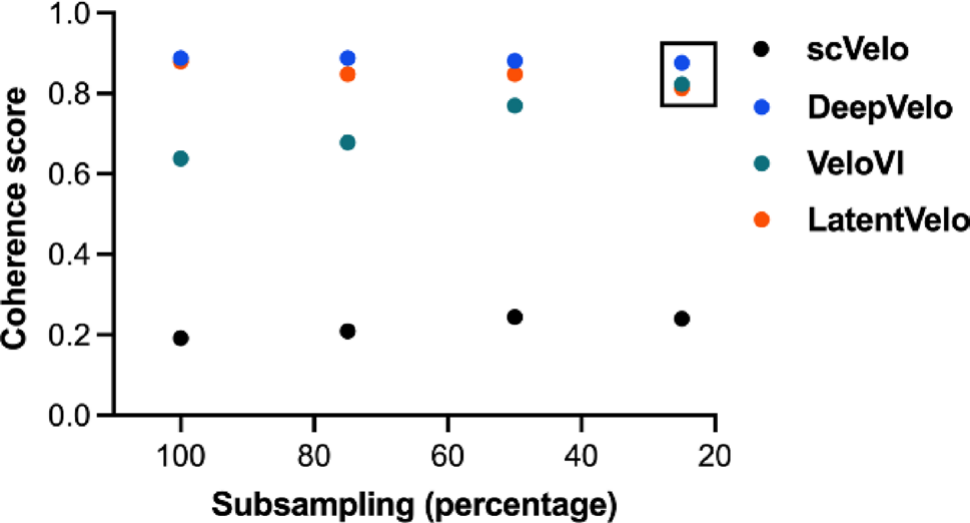
Analysis of predictive performance considering the coherence score in the downsamplings of the unspliced layer in the COVID-19 subgroup. X axis represents original data (100%) and the subsamplings (75%, 50%, and 25%). Y axis represents the values of the coherence score of the inferred RNA velocity vectors. Coherence scores range from 0 to 1, where values closer to 0 and 1.0 indicate random or noisy vector orientations and highly aligned, robust, and locally consistent vector fields within different unspliced fraction depths, respectively (details of values are presented in the Supplementary Table 2). The box depicts the coherence scores threshold (> 0.80) in samples with low unspliced fraction (25%)

The binomial downsampling of the unspliced layer (75%, 50%, and 25%) for the Covid-19 subgroup performed to access the stability under unspliced mRNA downsampling, showed divergencies comparing the analyzed tools. ScVelo, and DeepVelo coherence score kept relatively consistent across downsamplings, being the DeepVelo the most consistent tool. Interestingly, VeloVI highly increased the score across downsamplings (score from ~ 0.63 to ~ 0.82), while LatentVelo showed a slight reduction (from ~ 0.87 to ~ 0.81) (Fig. 7; Supplementary Table 2). Overall, deep learning models maintained higher coherence scores (> 0.80) at the most reduced unpliced genes fraction (25%), consistent with our other findings that they outperformed scVelo in reconstructing kinetic trajectories.

To expand on gene marker-based validation and uncover the biological relevance driving cell type transitions, we performed functional enrichment analyzes on the top velocity-driving genes of CD16 + NK cells, classical monocytes, and neutrophils of a human sample infected with the COVID-19 virus (part of GSE149689 dataset). The gene ontology enrichment analysis revealed differences in the biological resolution captured by the models. The scVelo and VeloVI provided more general or repetitive functional terms across all analyzed cell types, while DeepVelo and LatentVelo captured lineage-specific dynamic shifts. For instance, DeepVelo gene-drivers in the trajectories toward CD16 + NK cells were enriched for targeted effector functions (e.g., “cell killing” and “immune effector process”), the classical monocyte cluster was enriched for the “inflammatory response” and “response to stress”, and neutrophils were enriched with genes related to “leukocyte differentiation” and “leukocyte activation” (Supplementary Fig. 1).

Concerning the computational demands, the runtime and RAM consumption varied significantly by model, and sample size: (1) scVelo was the most efficient tool, requiring 2 to 7 min to finish the analysis and demanding low memory overhead (from ~ 9 to ~ 16 GB of RAM); (2) DeepVelo and VeloVI were the tools with the highest RAM demand, peaking at ~ 33 and ~ 35 GB of RAM, respectively; (3) LatentVelo and VeloVI were the slowest tools, demanding ~ 42 to ~ 168 min for inference, with VeloVI being the slowest one; (4) the running time and RAM consumption usually increased in function of the number of cells (Supplementary Table 1).

## Discussion

The present study focused on systematically comparing VAEs-based RNA-velocity methods (DeepVelo, VeloVI, and LatentVelo), and kinetic-based approach (scVelo) using two 10X Genomics scRNA-Seq datasets (GSE149689 and GSE203233). The following discussion interprets the main findings, addresses methodological and practical limitations, and provides recommendations to guide future applications and benchmarking of RNA-velocity inference methods.

### Comparative evaluation of RNA-velocity methods

Across both datasets (GSE149689 and GSE203233), VAE-based methods (DeepVelo, LatentVelo, and VeloVI) produced richer, stabler, and more spatially coherent and consistency velocity vectors than the classical scVelo model. The directional concordance measured by cosine similarity was significantly (p-value < 10^− 15^) higher in VAE-based approaches than in the scVelo method. Furthermore, cluster-wise velocity coherence scores corroborated our MSE findings, demonstrating that VAE-based approaches reduce global projection errors and also generate highly stable and biologically aligned vector fields within discrete cell states. Finally, the notably higher consistency scores from VAE-based models evidence that their vectors are biologically valid rather than mathematical artifacts of over-smoothing.

Overall, DeepVelo presented a narrow distribution and low overall error rate, while LatentVelo presented the highest absolute coherence scores globally. However, by combining visual UMAP inspections, cosine similarity distributions, MSE analysis, and velocity consistency score analysis, DeepVelo emerged as the most robust tool evaluated here. Altogether, DeepVelo offers an optimal balance between high local directional organization and global trajectory continuity, effectively recovering biological transitions without rigidly forcing vectors into artificially parallel local states.

### Empirical example: COVID-19 subgroup (GSE149689)

The COVID-19 subgroup illustrates the practical consequences of the methodological differences evaluated here. ScVelo generated relatively diffuse velocity vectors without a clear directional organization, whereas the VAEs (DeepVelo, LatentVelo, and VeloVI) produced sharper and more spatially consistent flows. In particular, DeepVelo achieved its highest cluster-wise coherence score (0.88) specifically within the COVID-19 subgroup, outperforming the other models.

A prominent example is the set of vectors that originate in neutrophil clusters and project toward NK-cell clusters. Marker inspection supports this inference because NK cells and neutrophils express distinct CD16 isoforms (CD16a in NK cells and CD16b in neutrophils), as previously described (Roberts and Barb 2018; Van Faassen et al. 2021). Similarly, velocity vectors that separate neutrophils from classical monocytes align with expected marker differences (CD16 versus CD14) reported in the literature (Silvestre-Roig et al. 2019; Ravenhill et al. 2020; Cormican and Griffin 2020).

The gene ontology enrichment analysis of the top velocity-driving genes of CD16 + NK cells, classical monocytes, and neutrophil cell types supported the hypothesis of an active transitional state concerning immunity during the severe COVID-19 response. Therefore, the gene ontology results corroborated the marker gene analysis, demonstrating that VAE-based approaches do not merely fit mathematical curves, but capture the underlying biological processes driving immune cell fate and function. Consistent with the functional profiles of cell-type analyzed here, the captured transitions fits the immunopathology described in recent large-scale COVID-19 single-cell atlases: the hyper-inflammatory transitions, emergency myelopoiesis, and dysregulated neutrophil and monocyte maturation pathways were characterized in foundational studies of this disease (Ren et al. 2021; Stephenson et al. 2021; Wilk et al. 2021), which was successfully recovered here by DeepVelo.

The concordances between inferred trajectories and canonical biological markers, and targeted functional ontologies increase confidence in VAE-derived results, particularly in infection-related contexts. In addition to the technical performance observed, it is suggested that VAEs may uncover transitional immune states relevant to inflammatory processes, with potential implications for understanding disease progression and immune dysregulation in viral infections.

### Validation via marker expression and limitations

The agreement between inferred directions and established marker expression (for example, CD16 isoforms and CD14/CD16 monocyte subsets) provides useful biological validation of velocity inferences. However, transcript abundance does not always directly reflect protein abundance or isoform processing (Cormican and Griffin 2020). Thus, interpretations based solely on transcriptomic markers remain provisional. Trajectory inferences should be corroborated with orthogonal biological measurements, such as flow cytometry, functional assays, or time-series data, when possible.

### Theoretical rationale for VAEs in single-cell trajectory inference

Several methodological features explain the apparent advantage of VAE-based approaches in complex single-cell contexts. VAEs learn compact, and non-linear latent representations that integrate multivariate gene-gene relationships and effectively attenuate sparse, and noisy measurements (Fu et al. 2025), enabling one to recover trajectories that gene-specific linear kinetic models may fail to detect. In addition, deep architectures scale to large high-dimensional datasets and automatically extract hierarchical features (Sharma et al. 2021), while autoencoders compress informative variance into latent spaces and filter irrelevant noise (Janiesch et al. 2021). These characteristics make VAE-based methods suitable for heterogeneous datasets, such as those analyzed in this study. Therefore, VAEs provide mathematical advantages and are in agreement with the growing trend to use deep generative models in computational biology to capture subtle variations and rare cellular states.

Regarding model interpretability, a frequent critique of deep learning methods is about their “black box” nature. ScVelo ranks genes based on their fit to a kinetic model, while deep learning tools utilize magnitude-based ranking within the inferred velocity layers. Our analysis showed that VAE-based models allowed for a better identification of specific genes driving velocity vectors; indeed, these highly-ranked genes were validated regarding their function via functional enrichment analysis. Altogether, despite the “black box” nature of deep learning models analyzed here, these models are able to rank genes that fits the biological relevance of studied phenomena, enabling researchers to pinpoint the specific molecular drivers of cellular transitions.

### Practical and computational constraints

Technical and computational limitations influenced performance and could restrict a broader application. Generating Velocyto loom files requires Cell Ranger BAM outputs and access to systems with at least 32 threads and 160 GB RAM. The absence of a local GPU substantially increased the training times for VAE-based methods, and LatentVelo could not be executed without GPU acceleration. Our computational benchmarking highlights the inherent trade-off between model accuracy and resource utilization. Although VAE-based methods require significantly more RAM (up to ~ 35 GB) and longer runtimes compared to scVelo, the substantial gain in vector consistency and robustness to data sparsity justifies the larger computational footprint.

To accommodate the mentioned constraints, our analyzes were restricted to the top 2,000 highly variable genes, allowing analysis without altering UMAP topology or the principal velocity patterns. However, sensitivity to lowly expressed or rare trajectory markers may be reduced using this approach. Therefore, one should approach interpretations of fine-grained or rare-state dynamics with caution.

The accuracy of splicing quantification is a fundamental limitation in velocity analysis. While scVelo velocity vectors became unreliable independent of unspliced fraction considering the confidence score, VAE-based frameworks maintained high directional coherence even at 25% downsampling. This data suggests that deep learning models may bypass the ‘low unspliced count’ bottleneck by leveraging global manifold information to stabilize the splicing signal. Therefore, we recommend that researchers evaluate splicing matrices before applying VAE frameworks, including visual inspection of the phase portraits of key trajectory-driving genes to ensure that they display canonical kinetic dynamics rather than artifactual noise. Additionally, comparing outputs from different pipelines and filtering genes with extreme or biologically implausible spliced/unspliced ratios could help mitigate downstream biases.

Beyond the constraints observed in our datasets, the scalability to atlas-level datasets (e.g., > 100,000 cells) presents an additional challenge for velocity inference. Despite that deep generative models inherently support mini-batch optimization, which theoretically could allow training on massive datasets without loading the entire matrix into RAM memory, the upstream preprocessing remains challenging. Generating spliced and unspliced count matrices for hundreds of thousands of cells demands a huge amount of RAM and storage using standard alignment-based pipelines. Future applications in atlas-scale data will likely require highly optimized alignment-free quantifiers or data sketching techniques to efficiently represent cellular diversity before VAE training.

The integration of RNA velocity with multi-omic data represents a critical frontier. Current velocity models are based exclusively on transcriptomic kinetics, which may not fully capture the regulatory delays between chromatin opening, transcription, and protein translation. Integrating paired single-cell epigenomic data (scATAC-seq) or surface protein expression (CITE-seq) could provide orthogonal validation and refine velocity vectors. Developing multi-modal generative architectures may prove essential to translate transcriptomic velocity inferences into a comprehensive perspective of cellular dynamics (Bergen et al. 2021; Qiu et al. 2022; Gorin et al. 2022; Weiler et al. 2024; Blair et al. 2025).

As mentioned, the preprocessing pipeline here used involved data normalization and log-transformation. These steps occur inplace in Scanpy, which means that the raw UMI counts in the AnnData.X matrix were transformed and lost. These transformations create an incompatibility with using scTour and SymVelo because they demand raw UMI counts and inputs inconsistent with log-normalized data, respectively. Modifying the data to use of scTour and SymVelo would introduce numerical instability, rounding errors, and confounding variables, and hence would promote artificial velocity artifacts or biased comparisons. Therefore, scTour and SymVelo were not included in the present study.

Automated cluster cell type annotations were particularly difficult for the GSE203233 dataset, which contained populations with few dominant cell types. Limited or inconsistent annotations challenge biological interpretation of inferred transitions: a velocity vector linking two clusters may represent a true developmental trajectory, a transient activation state, or an artifact resulting from mis-annotation or lower cell counts. Therefore, it is essential to use additional computational labels with marker-gene inspection and cross-validation against gold-standard datasets when possible. Establishing standardized annotation benchmarks would further improve the reproducibility of velocity analysis.

### Conclusions

VAE-based RNA-velocity methods provide improved directional concordance, local vector field coherence, and consistent vectors and trajectory continuity, particularly in complex infection-related contexts, as exemplified by the COVID-19 subgroup analyzed. Our results demonstrated that the higher performance of VAE-base methods is even robust to unspliced mRNA sparsity. These methodological advantages are accompanied by higher computational demands without harming the biological interpretability, even allowing for the identification of lineage-specific driver genes. Furthermore, the integration of magnitude-based gene ranking with functional enrichment provides a reliable framework for validating inferred cellular transitions against established molecular markers. We recommend here as practical guidelines: (1) to adequate the computational resources (RAM memory and GPU acceleration); (2) to conduct a sensitivity analysis on HVG selection and normalization strategies; (3) and to validate inferred directions using multimodal or protein-level data. For future applications, it is essential to perform benchmarking frameworks that incorporate multi-dataset, multi-omic, and cross-species comparisons to better define the efficiency of VAE-based methods.

## Supplementary Information

Below is the link to the electronic supplementary material.

Supplementary Material 1

Supplementary Material 2

## Data Availability

All codes used in this paper are available at https://gitlab.com/guilherme.valente/scrna-seq-velocity-benchmark.git.
