# Supplementary figures and images for: Comparison between a conventional tool and deep learning models for RNA velocity analysis of scRNA-Seq data
Source: Mol Genet Genomics. 2026 May 13;301(1):110. doi: 10.1007/s00438-026-02429-9 (PMC13171692; doi:10.1007/s00438-026-02429-9)

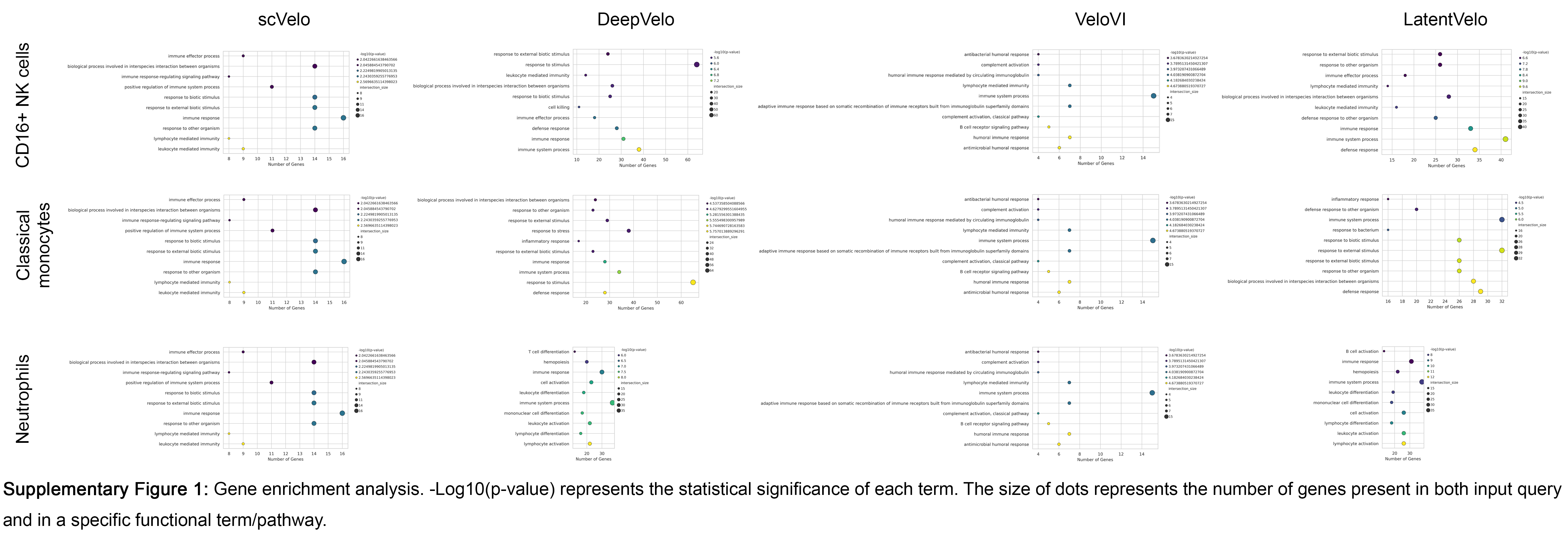

Supplement: Supplementary file 1 — Supplementary Material 1 [file 438_2026_2429_MOESM1_ESM.tif]
