# Supplementary material for: Comparison between a conventional tool and deep learning models for RNA velocity analysis of scRNA-Seq data
Source: Mol Genet Genomics. 2026 May 13;301(1):110. doi: 10.1007/s00438-026-02429-9 (PMC13171692; doi:10.1007/s00438-026-02429-9)
Supplement: Supplementary file 2 — Supplementary Material 2 [file 438_2026_2429_MOESM2_ESM.docx]

| **Sample** | **Tool** | **Mean confidence** | **Standard deviation** |
| --- | --- | --- | --- |
| Influenza | DeepVelo | 0.9208310195 | 0.08131106696 |
| Covid-19 | DeepVelo | 0.8882732 | 0.09105413758 |
| Control | DeepVelo | 0.9155195347 | 0.09575253413 |
| GF | DeepVelo | 0.808475275 | 0.1657726397 |
| ConvR | DeepVelo | 0.7890345386 | 0.1618005034 |
| Influenza | LatentVelo | 0.9612633678 | 0.05001988846 |
| Covid-19 | LatentVelo | 0.8798404356 | 0.08915864795 |
| Control | LatentVelo | 0.9630169867 | 0.04039404312 |
| GF | LatentVelo | 0.8401698535 | 0.1474607099 |
| ConvR | LatentVelo | 0.8155974108 | 0.1520435721 |
| Influenza | VeloVI | 0.8744185645 | 0.105115303 |
| Covid-19 | VeloVI | 0.6379198139 | 0.1888727907 |
| Control | VeloVI | 0.7436162589 | 0.1670078609 |
| GF | VeloVI | 0.7700000722 | 0.1236465072 |
| ConvR | VeloVI | 0.6724004079 | 0.1302691773 |
| Influenza | scVelo | 0.1830263284 | 0.1429479355 |
| Covid-19 | scVelo | 0.1923043185 | 0.1457747034 |
| Control | scVelo | 0.1985467427 | 0.1351354405 |
| GF | scVelo | 0.1933506189 | 0.1624933118 |
| ConvR | scVelo | 0.1922876239 | 0.1639695275 |

**Supplementary Table 1. Cluster-wise velocity consistency scores across samples.** The values represent the mean directional consistence and standard deviation of the inferred RNA velocity vectors for each sample across all models evaluated. The consistency score ranges from 0 to 1, where values closer to 0 and 1.0 indicate random or noisy vector orientations and highly aligned, consistent, and locally stable vector fields within biological clusters, respectively.

| **Subsampling** | **scVelo** | **DeepVelo** | **VeloVI** | **LatentVelo** |
| --- | --- | --- | --- | --- |
| 100% (original data) | 0.192304 | 0.888273 | 0.63792 | 0.87984 |
| 75% (subsampled) | 0.209978 | 0.888189 | 0.67788 | 0.847695 |
| 50% (subsampled) | 0.244381 | 0.881456 | 0.769694 | 0.847942 |
| 25% (subsampled) | 0.240232 | 0.875966 | 0.823277 | 0.812818 |

**Supplementary Table 2. Analysis of predictive performance considering the coherence score across downsampling of unspliced layer in the COVID-19 subgroup.** The values represent the directional coherence of the inferred RNA velocity vectors. Coherence scores range from 0 to 1, where values closer to 0 and 1.0 indicate random or noisy vector orientations and highly aligned, robust, and locally consistent vector fields within different unspliced read depths, respectively.

| **Tool** | **Sample** | **Sample size (num. of cells)** | **Runtime (min)** | **RAM memory consumption** |
| --- | --- | --- | --- | --- |
| DeepVelo | Control | 5,959 | ~7 | ~18GB |
| DeepVelo | Influenza | 11,632 | ~16 | ~28GB |
| DeepVelo | Covid | 13,388 | ~21 | ~33GB |
| DeepVelo | GF | 12,468 | ~19 | ~30GB |
| DeepVelo | ConvR | 9,466 | ~12 | ~24GB |
| LatentVelo | Control | 5,959 | *~42 | ~8GB |
| LatentVelo | Influenza | 11,632 | *~85 | ~12GB |
| LatentVelo | Covid | 13,388 | *~105 | ~12GB |
| LatentVelo | GF | 12,468 | *~111 | ~12GB |
| LatentVelo | ConvR | 9,466 | *~51 | ~10GB |
| VeloVI | Control | 5,959 | ~78 | ~21GB |
| VeloVI | Influenza | 11,632 | ~168 | ~30GB |
| VeloVI | Covid | 13,388 | ~145 | ~35GB |
| VeloVI | GF | 12,468 | ~155 | ~32GB |
| VeloVI | ConvR | 9,466 | ~126 | ~28GB |
| ScVelo | Control | 5,959 | ~2 | ~9GB |
| ScVelo | Influenza | 11,632 | ~4 | ~13GB |
| ScVelo | Covid | 13,388 | ~7 | ~16GB |
| ScVelo | GF | 12,468 | ~5 | ~14GB |
| ScVelo | ConvR | 9,466 | ~3 | ~10GB |

**Supplementary Table 3.** Computational resource utilization of tools analyzed here. *This parameter was estimated based on analyzes conducted in Google Colab utilizing a T4 GPU environment.
